# Evaluation of the amount of residual monomer released from different flowable composite resins

**DOI:** 10.1186/s12903-024-04005-2

**Published:** 2024-02-15

**Authors:** Didem Odabasi, Cigdem Guler, Didem Kucukaslan

**Affiliations:** 1 Faculty of Dentistry, Department of Pediatric Dentistry, Ordu University, Ordu, 52200 Turkey; 2 Pediatric Dentist, Ordu, 52200 Turkey

**Keywords:** Flowable composite, HPLC, Polymerization mode, Residual monomer

## Abstract

Today, resin materials are used in the restoration of permanent and deciduous teeth or as fissure sealants. The materials can contain different types of monomers (Bis-GMA, UDMA, TEGDMA). These monomers can be released into the oral cavity after polymerization. Residual monomers released from resin-containing restorative materials after polymerization have been reported to have negative effects on mechanical properties. The aim of our study is to evaluate the amount of residual monomers released after polymerization of different flowable composite resin materials using two different modes of LED light source. Composite disc samples (8 mm diameter/2 mm depth) prepared for each material group were polymerized using two different modes of the LED light device (Standard mode and extra power mode). HPLC (High Performance Liquid Chromatography) device was used to measure the amount of residual monomer release at 1 h, 1 day, 3 days and 7 days periods. Pairwise comparison of the differences between the materials was performed by Post-hoc test. For each residual monomer, the Kruskal Wallis test was used to analyze the difference between the materials in standard mode and the difference between the materials in extra power mode. According to the results of the study; Grandio flow flowable composite showed the highest release of TEGDMA and Bis-GMA while SDR® Flow flowable composite showed the lowest release of TEGDMA, Bis-GMA and UDMA. For all materials, the extra power mode resulted in more residual monomer release. TEGDMA and Bis-GMA release was detected in all tested flowable composites at all time periods.

## Introduction

Today, many different materials can be used for tooth restoration in pediatric dentistry. As an alternative to amalgam, the use of composite resin materials has increased rapidly as a result of increased aesthetic expectations and the development of adhesive materials [1]. One of the most important factors to reduce the difficulty of oral work in children is the ease of application of the restorative material. For this reason, flowable composite resins have become the preferred materials in pediatric dentistry.

Microleakage leads to a clinically unsuccessful restoration and increases the risk of secondary caries. Especially in class V and class II cavities, microleakage is difficult to detect and is known to occur below the enamel-cement boundary. Therefore, flowable composite resins are preferred over hybrid composites [2–4].

Flowable composites are hybrid resins with low filler content. With decreasing filler particle ratio, the viscosity decreased and the flow of the composite into narrow areas became easier. However, the high amount of matrix in these materials caused an increase in polymerization shrinkage and lower physical properties and durability [5, 6].

Resin-based dental materials used in modern dentistry are polymerized with light. Various light devices are used for polymerization. The most commonly used light devices are quartz-tungsten halogen devices [7]. The major disadvantage of halogen light devices is that they generate heat during active operation. Alternatively, LED light devices have been produced and reported to generate less heat during polymerization. In these systems, visible blue light is provided at a wavelength of 450–500 nm [8–12].

After polymerization, not all of the monomers in the composite materials are converted into polymer, and released into the oral environment. These residual monomers have been reported to disrupt the biological and mechanical structure of the material and reduce its durability [13–15]. In addition, residual monomers may cause cytotoxic, mutagenic, estrogenic effects or pulpal, gingival and oral mucosa reactions [16–18]. It is known that the amount of residual monomer release varies according to the different light devices used for polymerization and polymerization time [19, 20]. Studies using different power modes of the polymerization device are limited [21]. Therefore, in this study, two different power modes of the LED light device were used, considering that short working time would be an advantage in pediatric dentistry.

The residual monomers released after polymerization were mostly Urethane Dimethacrylate (UDMA), Bisphenol-A Glycidyl Methacrylate (Bis-GMA), Tri Ethylene Glycol Dimethacrylate (TEGDMA), 2-Hydroxyethyl Methacrylate (HEMA). The most suitable chromatographic methods for the analysis of these released monomers are High Performance Liquid Chromatography (HPLC) and Gas Chromatography/Mass Spectrometry (GC/MS). Reliable, reproducible, rapid and economical results have made this method the most preferred method [22, 23].

In the light of this information, the aim of our in vitro study is to evaluate the amount of residual monomers (TEGDMA, Bis-GMA and UDMA) released after polymerization of three different flowable composite resin materials using two different modes of LED light source (standard mode and extra power mode) as a function of time. The hypothesis tested was that there is no difference in the amount of residual monomers (TEGDMA, Bis-GMA and UDMA) released after polymerization of the flowable composite resins used in the study using two different modes of LED light source (standard mode and extra power mode).

## Materials and methods

In our study, in which the residual monomer release from three different flowable composite resins was examined according to different modes of the LED light device; preparation of test samples, HPLC analysis and statistical analysis were carried out respectively.

### Preparation of Test samples

Study groups were formed using three different flowable composite resins:

- Group 1: Grandio flow (VOCO, Cuxhaven, Germany).
- Group 2: Nova Compo HF (IMICRYL, Konya, Türkiye).
- Group 3: SDR™ (Smart Dentin Replacment) Flow (Dentsply Sirona, USA).

Table 1 presents the flowable composite resins tested and their properties.

**Table 1:** Flowable composite resins used in the study and their properties

Flowable Composite Resins	Organic Matrix	Filler Type.	Filler Rate %	Color	Manufacturer
Grandio flow	Bis-GMA, TEGDMA	Nanohybrid	80	A2	VOCO, Cuxhaven, Germany
Nova Compo HF	Bis-GMA, UDMA, TEGDMA	Nanohybrid	65–71	A2	IMICRYL, Konya, Türkiye
SDR™ (Smart Dentin Replacment) Flow	Modified UDMA, EBPADMA, TEGDMA	Bulk fill flowable	45	U	Dentsply Sirona, USA

In the study, 3 main groups of flowable composite resins and 2 subgroups were formed considering the polymerization mode (standard mode and extra power mode) in each group. The samples prepared in each subgroup were divided into 4 more subgroups according to the follow-up periods (1 h, 24 h, 3 days, 7 days). Power analysis was performed to determine the sample size in the study. Power analysis (G-power) was applied to set the number of samples for each group, taking into account the study of Cebe et al. Accordingly, the sample size was determined to be five in each follow-up period in the study (Power 0.99, n:5). A total of 120 samples were prepared, 40 samples of each flowable composite resin.

Plexiglass molds (8 mm in diameter and 2 mm deep) were used to prepare the samples to be tested in our study. First, a transparent matrix tape (ESR-P Universal strip) was placed on a glass sheet and plexiglass molds were placed on it. After the preferred restorative material was placed in the mold, the upper surface was again covered with transparent matrix tape. Strip tape was placed on the composite resin placed in the mold with hand tools and pressed with microscope glass to overflow the excess. The specimens were polymerized with VALO 3rd generation LED LCU (Ultradent Products Inc., South Jordan, UT, USA). In each use, a calibrated radiometer was used to check the output irradiance of the light source (TREE, model TR-P004, Foshan, Guangdong, China). When standard mode was used, light applications were performed 1 time for 20 s; when extra power mode was used, light applications were performed 2 times for 3 s each.

After light polymerization, the tranparent bands were removed. Finishing and polishing of the samples was performed with aluminum oxide (Al_2_O_3_) impregnated discs (Soft-Lex, 3 M Espe, St Paul, MN, USA) to remove the oxygen inhibition layer. Discs impregnated with aluminum oxide (Al_2_O_3_) were used respectively. Wet polishing was done when using discs.

### HPLC analysis

In the present study, pure chemicals of bisphenol A glycidyldimethacrylate (Bis-GMA), urethane dimethacrylate (UDMA) and triethylene glycol dimethacrylate (TEGDMA) (Sigma Aldrich, St. Louis, MO, USA) were used. In addition, 99.9% acetonitrile (Sigma Aldrich, 34,851) and 99.8% ethanol (Sigma Aldrich, 34,852) solutions were used. The prepared composite resin samples were placed in amber glass bottles containing 75% ethanol and 25% distilled water for extractions and kept in an oven at 37 °C between sampling sessions. For residual monomer release evaluation; TEGDMA, Bis-GMA and UDMA contents were analyzed. The residual monomer release amount of the samples was measured by HPLC device at 1 h, 24 h, 3 days and 7 days periods. The chemicals used in HPLC analysis in our study are given in Table 2.

**Table 2:** Chemicals used in HPLC analysis

Name of chemical used	Firm
TEGDMA	Sigma Aldrich, St Louis, MO, ABD
Bis-GMA	Sigma Aldrich, St Louis, MO, ABD
UDMA	Sigma Aldrich, St Louis, MO, ABD
%99,9 acetonitrile	Sigma Aldrich, 34,851
%99,8 ethanol	Sigma Aldrich, 34,852

**Fig. 1:**
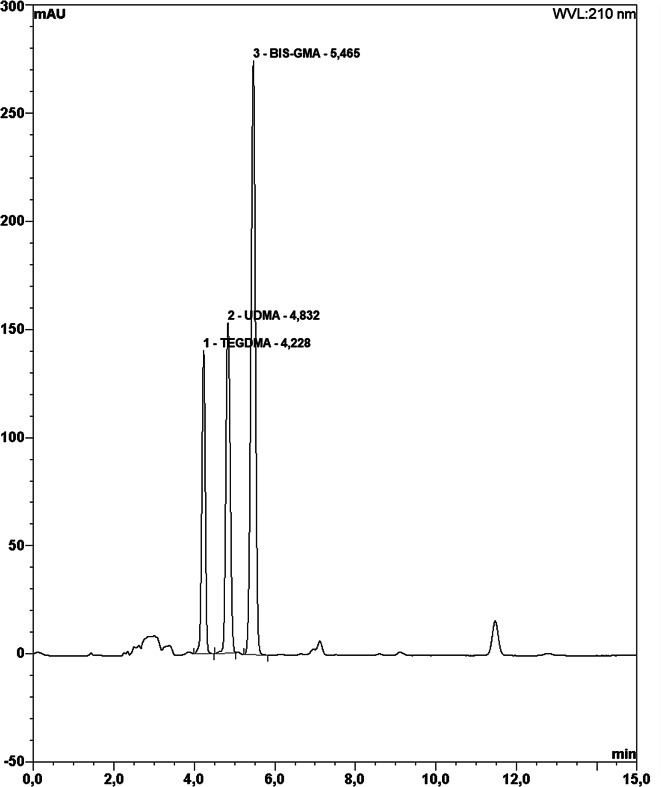
(1) The retention time of HPLC peaks of TEGDMA [4.22 min], (2) The retention time of HPLC peaks of UDMA [4.83 min], (3) The retention time of HPLC peaks of Bis-GMA [5.46 min]

**Fig. 2:**
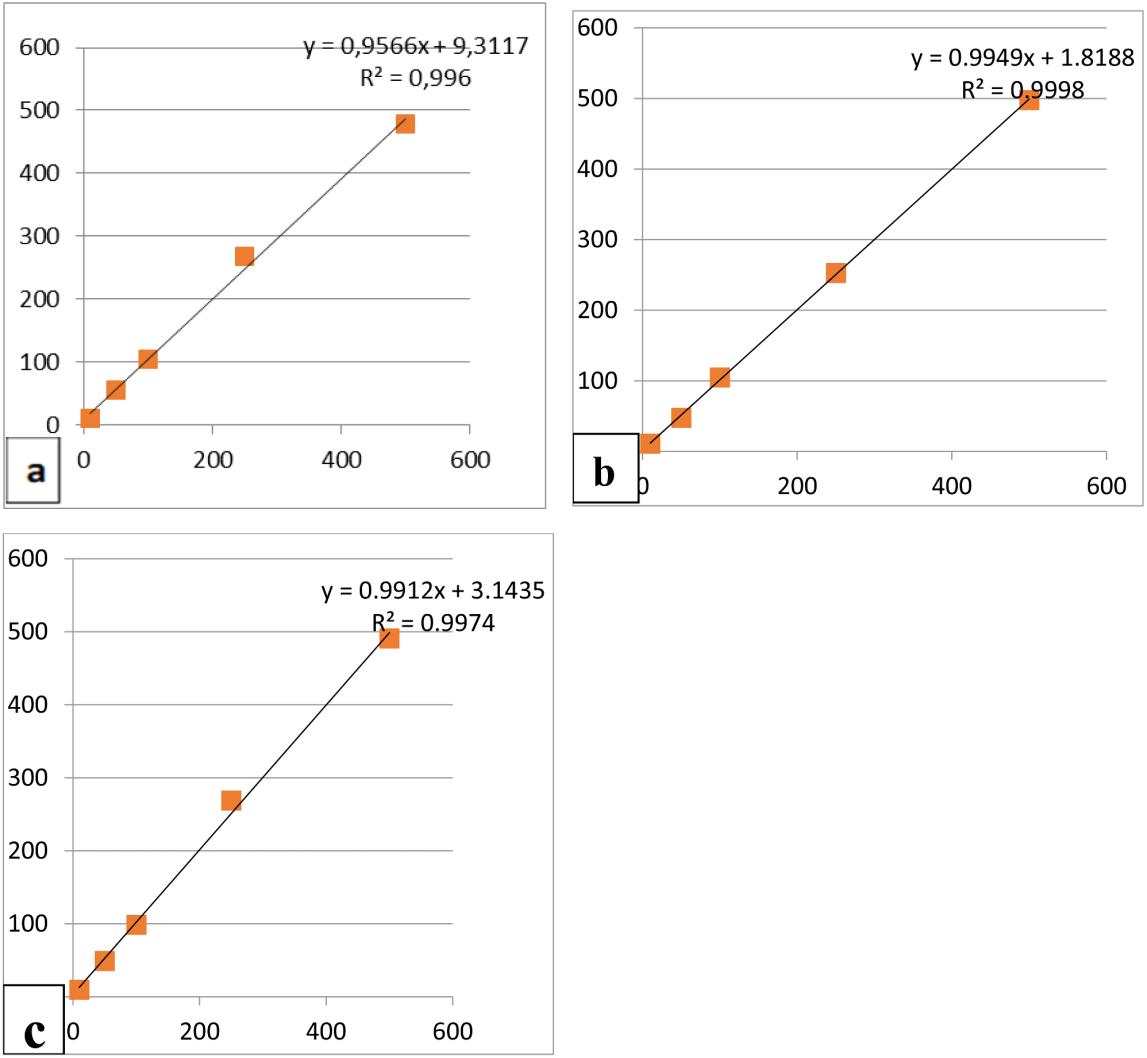
(**a**) HPLC calibration curves for TEGDMA (**b**) HPLC calibration curves for UDMA (**c**) HPLC calibration curves for Bis-GMA

Before HPLC analysis, standard solutions of monomers were diluted and introduced into the HPLC instrument to calibrate the instrument. As a result, retention times and peak values of monomers were determined (Fig. 1). Solutions of TEGDMA, UDMA and Bis-GMA monomers at 1, 10, 50, 100, 250 and 500 ppm were obtained and injected into the device. Retention times and peak values of the monomers were obtained by these procedures. Linear regression analysis of the concentrations in the standard solutions of the monomers was performed and the correlation coefficients and calibration equations of the monomers were obtained. The concentrations corresponding to the areas obtained in the chromatograms with the linear calibration equations were calculated in µg/ml (ppm). C18 reverse phase analytical column (particle size 5 μm, dimensions 25 cm x 4.6 mm) was used for HPLC Analysis. A single mobile phase of acetonitrile-water 80:20, v/v (isocratic mode) was used throughout the analysis. The injection volume was 20 µl and the flow rate was 1 ml/min. Chromotograms were obtained at 210 nm, the wavelength at which each monomer showed the best absorption (Fig. 2).

A 13 mm nylon syringe filter (Waters EDGE, USA) with 0.45 μm pore width was used to transport the samples. After filtration, the samples were placed in the HPLC device. At each time point, 1.5 mL solutions were taken from the vials containing the flowable composite resin samples using an eppendorf and transferred into 2 mL amber glass vials and injected into the HPLC device. Measurements were made on the chromatograms obtained. The amount of residual monomer released was determined by measuring the peak area. Accordingly, monomer analysis of the samples was finalized.

### Statistical evaluation

Statistical analysis was performed using Windows SPSS Statistics 22 (Statistical Package for Social Science, SPSS Inc., Chicago, IL, USA) software. One-way analysis of variance (ANOVA) test was applied to determine the differences between the test groups studied.

Pairwise comparisons of the differences between the materials were performed by Post-hoc test. For each residual monomer, the Kruskal Wallis test was used to analyze whether there was a difference between the materials in standard mode and whether there was a difference between the materials in extra power mode. In case of differences, pairwise comparisons were made with Mann Whitney U test. A value of *p* < 0.05 was accepted as a statistically significant difference.

## Result

There was a statistically significant difference between the release of TEGDMA, BisGMA and UDMA in all flowable composite resins tested (*p* < 0.01, Graph 1). Grandio Flow showed the highest release of TEGDMA (202.14 ppm) and BisGMA (334.25 ppm). Nova Compo HF showed the highest UDMA (217.97 ppm) release. SDR® Flow flowable composite showed the lowest release of BisGMA, UDMA and TEGDMA, respectively (7.62 ppm, 16.23 ppm, 127.28).

In the flowable composite resins tested, the extra power mode caused more monomer release for TEGDMA, Bis-GMA and UDMA release. However, this difference was not statistically significant only for UDMA release in SDR® Flow flowable composite (Table 3, *p* = 0,738).

**Table 3:** Distribution of TEGDMA, Bis-GMA and UDMA data by light source mode

Materials	TEGDMA			Bis-GMA			UDMA		
	Standard	Extra	p	Standard	Extra	p	Standard	Extra	p
Grandio flow	61,77 ± 16,45**a**	342,52 ± 49,24**A**	**< 0,001**	100,38 ± 44,90**a**	568,12 ± 83,54**A**	**< 0,001**	15,22 ± 29,14**a**	59,97 ± 23,17**A**	**< 0,001**
Nova Compo HF	12,86 ± 6,97**b**	239,64 ± 48,08**B**	**< 0,001**	15,44 ± 7,84**b**	233,38 ± 77,69**B**	**< 0,001**	25,72 ± 13,72**b**	410,23 ± 129,90**B**	**< 0,001**
SDR® Flow	58,78 ± 37,04**a**	195,79 ± 130,93**B**	**< 0,001**	4,66 ± 2,09**c**	10,57 ± 5,28**C**	**< 0,001**	8,30 ± 14,94**c**	24,16 ± 45,43**C**	0,738
p	**< 0,001**	**< 0,001**		**< 0,001**	**< 0,001**		**< 0,001**	**< 0,001**	

Different letters (a, b, c) indicate the statistical difference in residual monomer release between materials in standard mode in each column

Different letters (A, B, C) indicate the statistical difference in residual monomer release between materials in extra power mode in each column

**Fig. 3:**
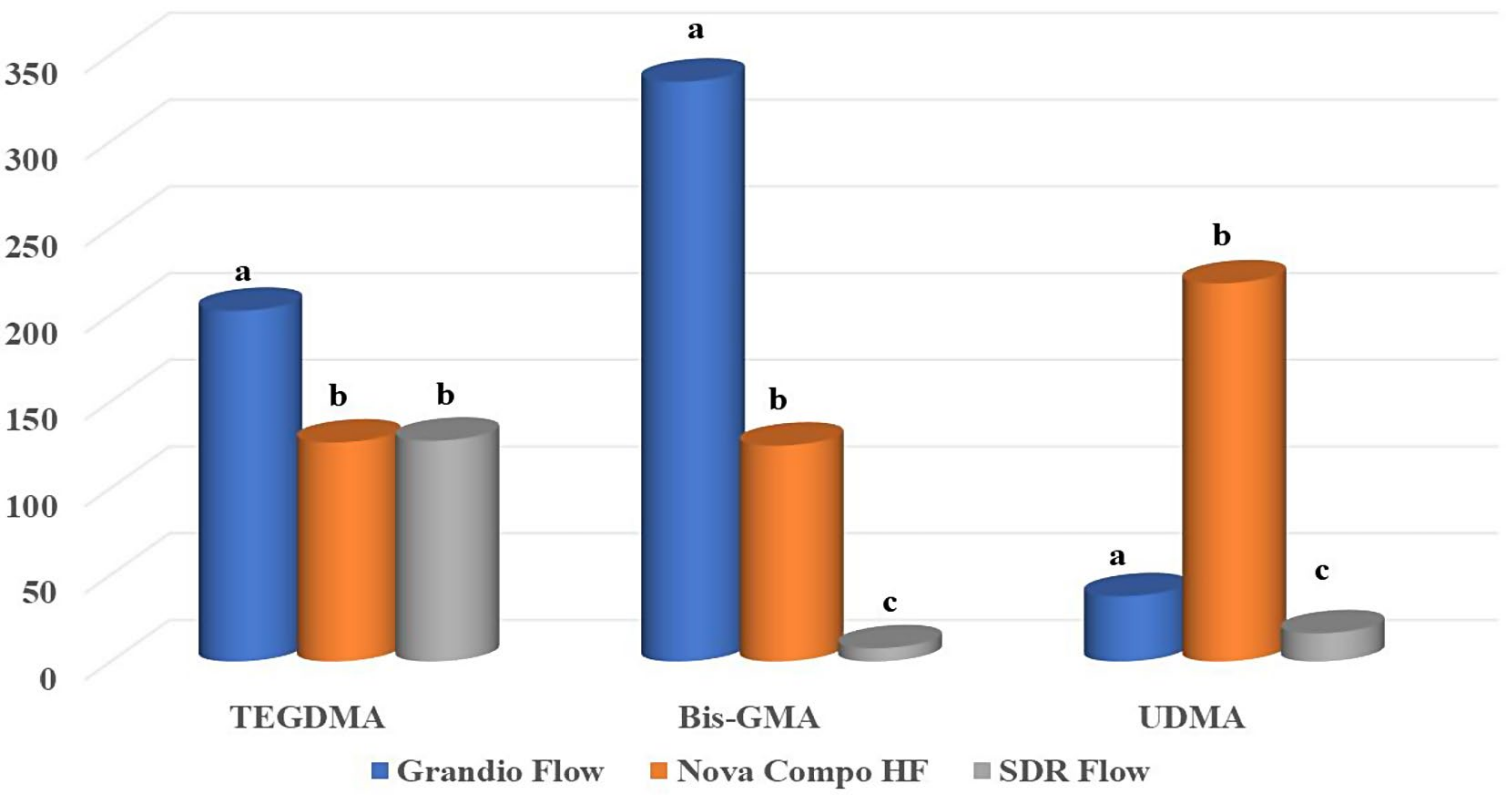
Distribution of TEGDMA, Bis‑GMA and UDMA data according to flowable composite materials. [Different letters (**a**, **b**, **c**) indicate the statistical difference in residual monomer release between materials in each column.]

**Fig. 4:**
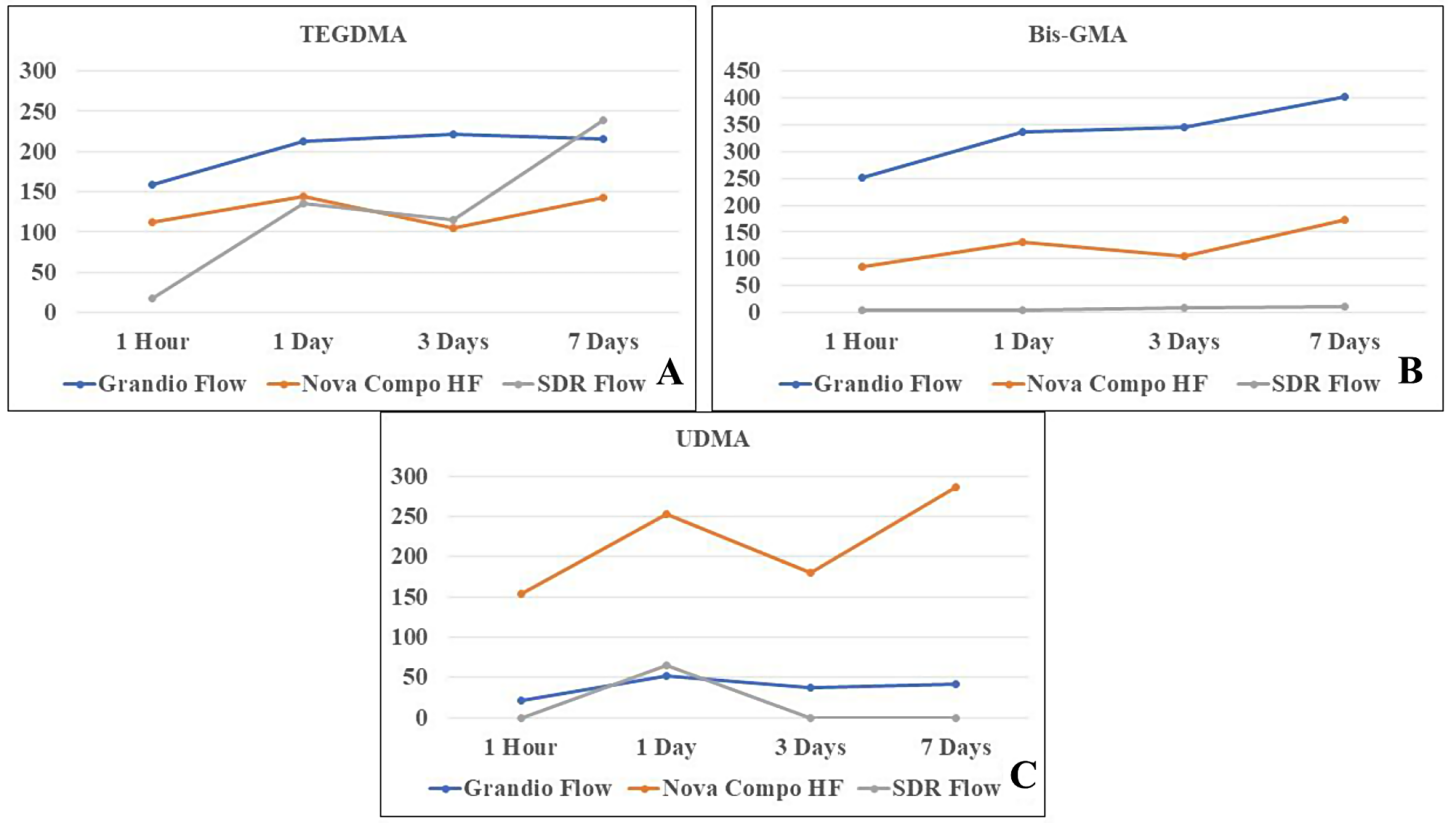
Distribution of residual monomer release according to follow-up periods. (**A**) TEGDMA, (**B**) Bıs-GMA and (**C**) UDMA

The distribution of TEGDMA, Bis‑GMA and UDMA data according to flowable composite materials is shown in Fig. 3. For TEGDMA release between the follow-up periods for each material, only SDR® Flow flowable composite showed a statistically significant difference (*p* < 0.001). Only in the 1-hour follow-up period, the difference between the materials in terms of TEGDMA release was found to be significant (*p* = 0.002). At 1 h, the least TEGDMA release was observed in SDR® Flow flowable composite (18.24 ppm) (Fig. 4A) **.**

There was a significant difference in Bis-GMA release between the materials in all follow-up periods (*p* < 0.001). In all follow-up periods, the lowest release was observed in the SDR® Flow flowable composite material in 1 day period (4.58 ppm) (Fig. 4B).

There was a statistically significant difference in the UDMA oscillation between the follow-up periods for each material (*p* < 0.001) only in the SDR flow flowable composite material. Except for the 1 day follow-up period, the difference between the materials in terms of UDMA release was found to be significant in all other follow-up periods (*p* < 0.001). In the 1-day follow-up period, the least UDMA release was observed in Grandio flow flowable composite (51.18 ppm), while in all other follow-up periods, the least UDMA release was observed in SDR® Flow flowable composite (Fig. 4C).

## Discussion

During the polymerization of light polymerized resin materials, carbon double bonds react and join the polymer chain. In the meantime, polymer monomer conversion is achieved with increasing viscosity. As a result of incomplete polymerization, the unreacted monomer is called ‘residual monomer’. As the degree of polymerization increases, the amount of unreacted residual monomer in the resin decreases and the physical properties of the restoration improve accordingly [24, 25]. In our study, the amount of residual monomers (TEGDMA, Bis-GMA and UDMA) released after polymerization of three different flowable composite resin materials using two different modes of third generation LED (standard mode and extra power mode) were evaluated as a function of time. The hypothesis that there is no difference between the amount of residual monomers (TEGDMA, Bis-GMA and UDMA) released after polymerization of the tested flowable composite resins using two different modes of third generation LED (standard mode and extra power mode) was tested. When the findings of our study were evaluated, the tested hypothesis was rejected.

HPLC (High Performance Liquid Chromatography) and GC (Gas Chromatography) are the most commonly used methods to determine the amount of residual monomers released by resin-based materials. It was stated that HPLC would be the most appropriate method for the determination of large molecular weight monomers [26]. Therefore, HPLC system was utilized to determine the residual monomer amounts in our study.

Halogen and LED light devices are frequently used for the polymerization of resin-based materials. Researchers have reported that the efficiency of polymerization depends on the light source used. While there are studies reporting that halogen light sources are more advantageous in terms of cytotoxicity than third-generation LEDs [27], there are also researchers stating that the light produced by LED light sources is sufficient for polymerization [28]. For this reason, third generation LED was preferred for polymerization in our study. Cender et al. used the light devices used in our study in their study in 2021 [29].

It is known that resin materials should be irradiated in 2 mm layers to increase the polymerization efficiency [30, 31]. Molds of different sizes (0.5, 1, 2, 4, 6 mm) and materials (teflon, stainless steel, silicone, brass, rubber) were used in the studies [32–37]. In this study, samples of flowable composite resins were prepared using pre-prepared 2 mm high, 8 mm diameter Plexiglas molds. Polishing disks were used to remove the oxygen inhibition layer of the obtained samples. Oxygen inhibition layer is known to contain residual monomer. By polishing the surfaces, standardization of the samples was achieved in determining the residual monomer release.

The solutions in which the materials were kept for the analysis of monomer release varied. The materials used include artificial saliva [38], cell culture medium [39], acetonitrile [40], distilled water [41], deionized water [42], ethanol-water mixture [43]. The United States Federal Drug Administration (FDA) recommends a 75% ethanol-water solution, which has been used in several studies, as a clinical food-oral simulant liquid [44, 45]. Ethanol enables the release of residual monomers that remain unreacted in the polymer matrix [21, 22]. Therefore, 75% ethanol-water solution was used in our study.

The gold standard for composite resins is that the polymerization time should be 40 s and the distance between the light source and the top surface should not exceed 2 mm [46]. In a study by Sideridou and Achilias, Bis-GMA, TEGDMA, UDMA and Bis-EMA resin monomers were polymerized in Teflon molds for 60 s, 80 and 100 s and then kept in ethanol-water mixture and they observed that the amount of residual monomer decreased with increasing polymerization time. This finding is consistent with the findings of our study [21]. In our study, the irradiation time was 20 s in standard mode and 3 s twice in extra power mode in accordance with the manufacturer’s recommendations, and when the findings obtained were evaluated, more monomer release was detected in extra power mode in the release of TEGDMA, BISGMA and UDMA in the flowable composite resins tested. As Gonulol et al. stated in their study; shorter polymerization times are often preferred in clinical conditions [47]. Especially when working with pediatric patients, it can be aimed to use extra power mode to shorten the polymerization time and to ensure that the procedures are completed in a shorter time by maintaining cooperation. However, it is also important to consider the residual monomer release after the polymerization process to be used.

The most commonly used monomers in composite resins are Bis-GMA, Bis-EMA, UDMA and TEGDMA. This information was taken into consideration when determining the flowable composite resins included in the study. For the evaluation of residual monomer release in our study; TEGDMA, Bis-GMA and UDMA contents were analyzed. In a study evaluating the release of Bis-GMA, TEGDMA, UDMA and Bis-EMA monomers; Bis-GMA was observed to have the highest release value among all monomers [22]. In a study conducted using three different colored compomer materials, HEMA, BisGMA, TEGDMA, UDMA monomer release was compared in 11 different time periods after polymerization and it was concluded that the monomer with the highest release value was Bis-GMA [36]. In our study, Bis-GMA was found to be the residual monomer with the highest release in accordance with this finding.

Caughman et al. correlated the cytotoxic potential of resin composites on primary human gingival fibroblast culture with the degree of monomer conversion of three composite materials with different filler ratios ranging between 45% and 88%. It has been reported that cellular toxicity decreases as the percentage of monomer conversion increases [48]. In our study, three different flowable composites with filler ratios between 45 and 80% were tested. As a result, a statistically significant difference was found between the release of TEGDMA, BisGMA and UDMA in all flowable composite resins tested. This may be due to the difference in filler ratios of flowable composite resins.

Kavahara et al. investigated the amount of residual monomer released from temporary resin materials and determined the measurement periods as 1st, 3rd, 6th, 24th hour and 3rd, 7th, 14th day, Miletic et al. investigated the residual monomer release from adhesives and determined the measurement periods as 1st, 6th, 24th, 96th hour and 7th day [49, 50]. Considering all these, in our study, similar to the studies of Lagocka et al. ; the measurement periods of residual monomer release were determined as 1 h, 1 day, 3 days and 7 days [17].

It has been reported that monomer release from resin materials is high in the first minutes and this amount decreases over time [22]. Researchers have stated that monomer release continues for 24 h [51]. In our study, unlike these studies, it was observed that residual monomer release was detected in all time periods (1 h, 24 h, 3 days and 7 days) even though increases and decreases were observed in all residual monomer types evaluated. The difference may be due to the different restorative materials used or the different follow-up periods.

When the residual monomer releases among the composites tested in our study were analyzed; a statistically significant difference was found only in the 1-hour time period for TEGDMA (*p* < 0.002), in all time periods for Bis-GMA (*p* < 0.001) and in all time periods except 1 day for UDMA (*p* < 0.001). The lowest residual monomer release was detected in SDR Flow material, which is a bulk fill flowable composite (1 h for TEGDMA and 1 day for Bis-GMA). UDMA was only observed at 24 h time period in SDR Flow material which is a bulk fill flowable composite. TEGDMA is a monomer that reduces viscosity. The early increase in TEGDMA compared to Bis-GMA can be explained by the fact that TEGDMA diffuses faster due to its higher ratio and its molecular weight is lower than other monomers.

Several studies have demonstrated that the resıdual monomers exhibit systemic and local toxic properties, including cytotoxic, genotoxic, mutagenic, and allergenic effects. These studies have indicated that the cytotoxicity ranking of these basic monomers is BisGMA > UDMA > TEGDMA [52–55]. Noda et al. in their study examining the effects of dental resins on THP-1 human monocytes, determined the cytotoxic effect value of TEGDMA as 4000µM (1144 ppm) [56]. In our study, the TEGDMA release value of all flowable composites tested was found below 1144 ppm. In a study examining the cytotoxicity of dental composites on human gingival fibroblasts, the cytotoxicity value for UDMA was determined as 0.2 mmol/l (94.11 ppm) [57]. In our study, values below 94.11 ppm were obtained in the other flowable composites tested, except for Nova Compo HF flowable composite.

Our study is an in vitro study. The chemical structure of the patient’s oral flowable composite resins, stresses on the restoration, the amount of wear and polymerization processes are effective in the release of residual monomer under in vivo conditions. The monomer content of the materials used and their relationship with each other also have an effect. Laboratory conditions may not fully mimic the clinical situation in in vivo conditions and may not fully reflect the monomer release values. For these reasons, the amount of residual monomers released from resin materials and their biological effects on tissues should be supported by in vivo studies.

This study has some limitations. Firstly, only three different flowable composite resins were tested. The findings may vary when different types of adhesive materials are used. Secondly, the standard and extra power modes of a third generation LED for polymerization were tested. The findings may vary when different light sources or different polymerization times are used. Finally, 1 h, 24 h, 3 days and 7 days were used as follow-up periods in our study. The findings may vary when different follow-up periods are used.

## Conclusion

Within the limits of our study, the following results were obtained:

1. Among the tested composites, the lowest residual monomer release was determined in the bulk fill flowable composite.
2. In all composites, the amount of residual monomer released after polymerization in the extra power mode of the light device was higher compared to the standard mode.

## Data Availability

Personal data of the participant will not be shared. The data used and/or analyzed during the current study are available from the corresponding author on reasonable request.

## Abbreviations

HPLC: High Performance Liquid Chromatography
